# Case Presentation: Functional Assessment of a *CASR* Variant Identified in a Patient with Hypercalcaemia Confirms Familial Hypocalciuric Hypercalcaemia in the Patient and a Sister Previously Misdiagnosed with Primary Hyperparathyroidism

**DOI:** 10.1155/2024/6652801

**Published:** 2024-02-03

**Authors:** Bryan K. Ward, Kirsten A. Loffell, John P. Walsh, Warwick D. Howe, Suzanne J. Brown, Scott G. Wilson

**Affiliations:** ^1^Department of Endocrinology and Diabetes, Sir Charles Gairdner Hospital, Nedlands, WA, Australia; ^2^Harry Perkins Institute of Medical Research, Centre for Medical Research, QEII Medical Centre, University of Western Australia, Nedlands, WA, Australia; ^3^Medical School, University of Western Australia, Nedlands, WA, Australia; ^4^Department of General Medicine, Joondalup Health Campus, Ramsay Health, Joondalup, WA, Australia; ^5^School of Biomedical Sciences, University of Western Australia, Nedlands, WA, Australia; ^6^Department of Twin Research and Genetic Epidemiology, King's College London, London, UK

## Abstract

**Background:**

Primary hyperparathyroidism (PHPT) and familial hypocalciuric hypercalcaemia (FHH) are common causes of hypercalcaemia. Patients are mostly asymptomatic in the case of FHH and often so in the case of PHPT. In addition, biochemical parameters show considerable overlap, making differential diagnosis difficult. Genetic screening for inactivating variants in the calcium-sensing receptor (*CASR*) gene that are causative of FHH assists with the diagnosis since such variants are not generally associated with PHPT. However, novel *CASR* variants must undergo functional assessment before they can be definitively assigned a causative role in FHH. *Case Presentations*. We describe a 73-year-old female (patient A) who presented with mild parathyroid hormone (PTH)-dependent hypercalcaemia and a history of osteoporosis. Family history revealed that her sister (patient B) had presented a decade earlier with symptoms of PHPT including a history of mild hypercalcaemia and multiple renal calculi, prompting parathyroid surgery. However, a subtotal parathyroidectomy did not resolve her hypercalcaemia long term. On this basis, genetic screening was performed on patient A. This identified a heterozygous variant in the *CASR*, NM_000388.4:c.T101C: p.Leu34Pro (L34P). Functional analysis showed that the L34P variant was unable to produce mature, dimerized receptor and did not respond to Ca^++^ ions. Adopting American College of Medical Genetics-based guidelines, the variant was classified as 'Pathogenic (II)'. Patient B was subsequently found to carry the L34P variant heterozygously, confirming a diagnosis of FHH, not PHPT.

**Conclusion:**

This study shows the importance of examining patient's family history in providing clues to the diagnosis in isolated cases of hypercalcaemia. In this case, history of a sister's unsuccessful parathyroidectomy prompted genetic screening in a patient who might otherwise have undergone inappropriate parathyroid surgery. Screening detected an inactivating *CASR* variant, firming up a diagnosis of FHH. These studies reaffirm the requirement for functionally assessing novel *CASR* variants prior to assigning causality to FHH.

## 1. Background

Hypercalcaemia is a common finding in both primary care and hospital settings [1]. It is classified as either parathyroid hormone (PTH)-dependent, comprising primary hyperparathyroidism (PHPT) (occurring sporadically or as part of familial syndromes including multiple endocrine neoplasia 1, 2a, or 4 and familial-isolated primary hyperparathyroidism), tertiary hyperparathyroidism and familial hypocalciuric hypercalcaemia (FHH), or PTH-independent, caused by malignancy or less commonly by other disorders such as thyrotoxicosis, granulomatous disease, or vitamin D intoxication [1]. PHPT is usually caused by an adenoma or hyperplasia in one or more of the parathyroid glands leading to overproduction of PTH which in turn causes an elevation in serum calcium levels. PHPT is often asymptomatic and may be diagnosed incidentally during routine biochemical analyses initiated for other reasons. Symptoms in mild cases tend to be nonspecific and may include joint aches and pains, fatigue, weakness, loss of appetite, difficulty concentrating, and mild depression. More severe cases may exhibit significant loss of appetite, constipation, nausea, polydipsia, and/or frequent urination. Subsequently, long-term loss of calcium from bone may lead to osteoporosis, while increased calcium levels in blood may result in impaired kidney function and/or nephrolithiasis. Surgery to remove the involved parathyroid gland(s) is therefore recommended in many cases, although mild, asymptomatic PHPT can be managed conservatively (for review, see Bilezikian et al. [2]). FHH is most often caused by autosomal dominantly inherited inactivating (usually missense) variants of the calcium-sensing receptor (*CASR*) gene that result in an elevated set point for calcium-mediated PTH suppression and renal tubular calcium reabsorption [3–6]. As a result, serum calcium is mildly elevated and urine calcium inappropriately low, with unsuppressed PTH levels that are generally within the upper reference range or mildly elevated. Hypercalcaemia in FHH patients is lifelong but generally does not result in obvious disease symptoms or long-term sequelae [3–5], and parathyroid surgery is not indicated. In the clinic, it is important to distinguish between PHPT and FHH in order to avoid inappropriate parathyroid surgery in cases of FHH [7, 8]. However, this presents a problem if using biochemical criteria alone as the two disorders can show considerable overlap biochemically [7–9]. Genetic evaluation of the *CASR* in patients in whom the diagnosis of PHPT/FHH is not clear is therefore recommended since inactivating variants in the *CASR* are known to be causative of FHH but not PHPT. However, not all variants of the *CASR* are inactivating and the presence of a novel, missense variant alone is not sufficient to assign causality to FHH; bioinformatics analysis together with functional studies to prove that a novel variant is inactivating is considered a fundamental requirement according to the American College of Medical Genetics (ACMG) guidelines [10].

## 2. Case Presentations

During 2019, a 73-year-old female (patient A) presented for assessment of hypercalcaemia. She had a history of osteoporosis with a previous fractured left radius (2013) and fractured right radius (2015) following minimal trauma. Following the second fracture, bone density measured by dual-energy X-ray absorptiometry (DXA) gave the following results: lumbar spine *T* score: −1.6, femoral neck *T* score: −2.1, and total hip *T* score: −2.5. She was commenced on denosumab (60 mg every 6 months) which she was still taking at the time of assessment; she was also taking a vitamin D supplement but no calcium supplementation. Biochemical assessment revealed mild hypercalcaemia with unsuppressed PTH consistent with either PHPT or FHH, although the spot urine calcium/creatinine clearance ratio (<0.01) and calcium excretion values favoured a diagnosis of FHH (Table 1). Dynamic four-dimensional computed tomography (4D CT) of the parathyroids (arranged by her primary care physician) did not identify a parathyroid adenoma, and renal tract ultrasound was normal with no evidence of calculus or nephrocalcinosis.

It was noted that the patient's sister (patient B) had presented a decade earlier in April 2009, aged 58, with lethargy, widespread body pains, proximal myopathy, and low mood and was found to have mild ionized hypercalcaemia, high-normal total calcium (albumin-corrected) with unsuppressed PTH, and hypocalciuria (Table 1). Renal tract CT at that time showed a tiny nonobstructing calculus at the lower end of the right ureter, and this was successfully removed at ureteroscopy. Subsequent cystoscopy revealed additional renal calculi which were also removed. Bone density testing was undertaken with normal results. Parathyroid scintigraphy using 99m-Tc-sestamibi did not localise an adenoma. Bilateral neck exploration surgery was performed. Intraoperatively, the right superior parathyroid and left inferior parathyroid appeared slightly bulky and were removed, whereas the right inferior gland appeared macroscopically normal and was retained. The left superior parathyroid was not identified. The two resected parathyroids were reported as histopathologically normal with no evidence of adenoma. Postoperatively, the patient's calcium levels were essentially unchanged with persistent ionized hypercalcaemia.

**Figure 1:**
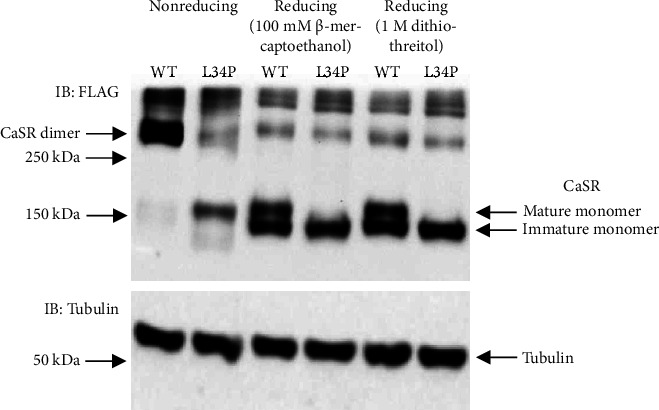
Western blot analysis of wild type and L34P variant CaSR examined under nonreducing and reducing conditions. HEK293 cells were transfected with wild type or variant CaSR-FLAG-tagged plasmid (5 *µ*g) and then incubated for 48 hrs, and the cells were then lysed, and 80 *µ*g protein, with or without a reducing agent, was separated by SDS-PAGE. The gel was blotted on to nitrocellulose and then probed with mouse monoclonal antibody to FLAG-tag (upper panel) and *α*-tubulin (as a loading standard; lower panel), followed by goat antimouse HRP-conjugated secondary antibody. Protein bands were visualized using ECL chemiluminescence reagent in a Bio-Rad Molecular Imager. Experimental details are described in reference 18. The image is representative of several independent experiments. The FLAG and tubulin immunodetections were derived from the same blot. Cropping was used to ensure a more concise presentation of the gel image (see Supplementary Figures 1 and 2).

**Figure 2:**
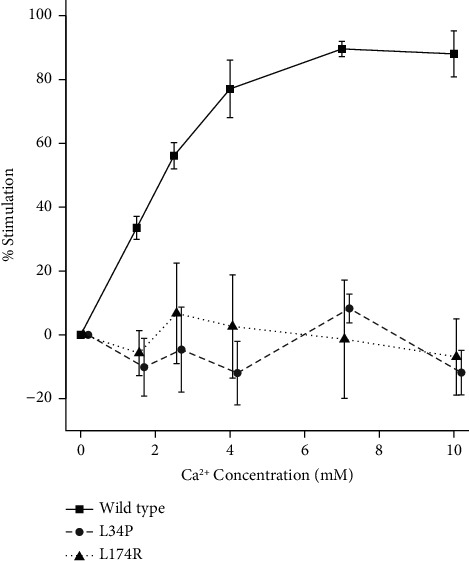
Dose response curves for wild type, L34P variant, and a known inactivating (L174R) variant CaSR using the IP-One ELISA assay following Ca^++^ ion (1.5, 2.5, 4, 7, or 10 mM) stimulation in HEK293 cells. The IP-One ELISA assay was performed as described previously (see reference 18). Values represent the mean % stimulation (with ±2x standard error of the mean error bars) plotted against Ca^++^ ion concentration for three separate experiments. Calculation of % stimulation and statistical comparisons between the wild type and variant receptors were as described in reference 18.

Based on the somewhat ambiguous biochemical parameters and the patient's sister's apparently unsuccessful parathyroidectomy, FHH was considered the more likely diagnosis for patient A, so a blood sample was submitted for genetic testing for defects in the *CASR* gene. DNA was extracted, and segments of the *CASR* were amplified by PCR and underwent Sanger sequence analysis for variants as previously described [11]. This revealed a heterozygous missense variant in exon 2 of the *CASR*, NM_000388.4:c.T101C:p.Leu34Pro (L34P), affecting the amino-terminal end of the Venus flytrap “sensing” domain of the receptor. This variant, designated rs1559955362, has been reported in ClinVar as a variant of unknown significance. Although our bioinformatics analysis using ANNOVAR and Ensembl software tools [12, 13] revealed that the variant is likely to be deleterious/damaging at least by most criteria (Table 2), application of the more conservative University of Maryland Genetic Variant Interpretation Tool, which is based on ACMG guidelines [10], also classified the variant as one of unknown significance, largely due to the lack of functional data demonstrating that the variant is inactivating. We then set out to perform the required functional studies by cloning the variant in the mammalian expression vector, pcDNA3.1, and expressing it in HEK293 cells (which do not endogenously express CaSR) and comparing its expression with wild type receptor by Western blot analysis performed either under nonreducing or reducing conditions [14]. Under nonreducing conditions, the wild type receptor was observed to dimerize as expected; however, the variant demonstrated little if any ability to dimerize, whereas under reducing conditions, the wild type receptor showed both immature monomeric receptor (140 kDa) and mature glycosylated monomeric receptor (160 kDa) as expected, but the L34P variant demonstrated only the immature form of the receptor monomer (Figure 1, Supplementary Figures 1 and 2; supplementary figures represent uncropped immunoblots that comprise Figure 1). Since a mature, dimerized receptor is essential for CaSR signalling [15, 16], these results suggested that the L34P variant would be devoid of signalling capacity; i.e., the receptor would be inactive. This was confirmed by performing an IP-One ELISA assay comparing Ca^++^-mediated D-myo-inositol-1-phosphate accumulation for wild type and variant receptors as described previously [17, 18]. A typical dose response accumulation of D-myo-inositol-1-phosphate was observed for the wild type receptor; however, the L34P variant showed no significant response to Ca^++^ at any concentration of Ca^++^ ions (*p* < 0.01) and behaved similarly to a known inactivating receptor, L174R, which was used as a positive control for defective receptor function (Figure 2). With the subsequent inclusion of these functional analysis results, application of the University of Maryland Genetic Variant Interpretation Tool reclassified the variant as 'Pathogenic (II)'.

Patient B was then approached to supply a blood sample for genetic testing. Sequence analysis of the relevant segment of the *CASR* revealed the heterozygous presence of the L34P variant.

## 3. Discussion

We examined a patient (patient A) with hypercalcaemia but with only minimal symptoms that might relate to PHPT, apart from her osteoporosis which would be considered an advanced symptom and not necessarily one that is specific to PHPT. In addition, her biochemical profile was more in keeping with FHH with mild hypercalcaemia, PTH in the normal range or only slightly elevated, and having low urine calcium excretion (Table 1). Her calcium/creatinine clearance ratio averaged 0.004, well below the 0.01 cutoff, suggesting FHH [7, 9, 19]. There was a history of hypercalcaemia and kidney stones in the family with her sister undergoing an apparently unsuccessful parathyroidectomy some years earlier with no consistent change in biochemical parameters postsurgery (Table 1). On this basis, we performed genetic studies to determine whether alterations in the *CASR* gene might firm up the provisional diagnosis of FHH. We identified a missense nucleotide variant in the *CASR* (NM_000388.4:c.T101C) which results in a leucine to proline substitution at amino residue 34 of the receptor (L34P). Bioinformatics analysis using a number of parameters suggested that the variant may be deleterious; however, not all the bioinformatics indices were completely aligned. For example, the combined annotation-dependent depletion (CADD) score, which is a widely used composite metric that integrates a diverse range of genome annotations, yielded a CADD Phred score of 27.6 (i.e., falls within the top < 1% of deleterious variants in the genome); however, for conservative interpretation of this metric, Ensembl recommends a threshold score of 30 (i.e., falls within the top < 0.1% of deleterious variants in the genome) and this variant does not achieve that score (13). On this basis, applying the genetic variation interpretation tool, the variant could only be classified as one of unknown significance. Further studies showed that the variant was defective in maturation and was unable to dimerize, and functional studies using the IP-One assay confirmed that it is an inactivating variant; i.e., the accumulation of D-myoinositol-1-phosphate is completely abrogated. D-myoinositol-1-phosphate is a downstream metabolite of G_q/11_-dependent activation of phosphoinositide-specific phospholipase C, a pathway shown to play a critical role in Ca^++^-mediated control of PTH secretion [20]. Subsequent bioinformatics analysis using the genetic variation interpretation tool enabled reclassification of the variant as 'Pathogenic (II)' and therefore reportable as causative of FHH in this patient.

Patient A's sister (patient B) was provisionally diagnosed with PHPT a decade earlier. Her hypercalcaemia was mild and accompanied by hypocalciuria, but there was no family history of hypercalcaemia at that time, and she had a history of renal calculi which strongly influenced the decision to perform parathyroid exploration. Although only 3 rather than 4 parathyroids were identified during surgery, there was no convincing evidence of adenoma or hyperplasia and ionized hypercalcaemia persisted postoperatively. Although it was some years later, we were able to perform genetic analysis of the *CASR* from patient B and she was found to have the L34P variant and therefore can be confirmed as having FHH rather than PHPT. These results demonstrate the importance of evaluating family history in determining the appropriateness of genetic screening for patients with hypercalcaemia, particularly those presenting with mild or nonspecific symptoms and a biochemical profile difficult to distinguish between PHPT and FHH. While variants in the *CASR* gene account for approximately 65% of the cases of FHH (FHH-1) and should first be examined, other genes, namely, the G-protein subunit *α*11 gene (*GNA11*) and the adaptor-related protein complex 2 sigma 1 subunit gene (*AP2S1*), should also be considered in the event of negative findings for the *CASR*. Although rare compared to variants in the *CASR*, variants in *GNA11* and *AP2S1* give rise to FHH-2 and FHH-3, respectively [6, 21, 22].

The importance of genetic screening for FHH is highlighted by a number of studies demonstrating that there is no clear-cut distinction between FHH and PHPT based on biochemical parameters alone. Early studies demonstrated that serum PTH together with fasting calcium excretion were able to distinguish FHH from PHPT; however, this was based on a rather small number of FHH cases from four families [8]. In another study, approximately 20% of the individuals with surgically proven PHPT were found to have preoperative intact serum PTH levels within the upper limit of the reference range [23] while 24-hr renal calcium excretion is not always a good predictor of FHH with more than 50% of the FHH patients in one study having a normal or elevated calcium excretion [9]. The calcium creatinine clearance ratio (CCCR) is generally accepted as the best parameter in distinguishing FHH from PHPT with values <0.01 indicative of FHH and values >0.02 indicative of PHPT with a “gray” area in between [7, 9, 19, 24]. However, even this parameter has its limitations. In a recent study, two thirds of the patients demonstrated a CCCR <0.02 even though they had histologically confirmed PHPT and over 40% of the FHH patients presented with CCCR readings >0.01 [9]. In order to increase specificity, some clinicians have advocated genetic testing for FHH where CCCR is <0.02 [24, 25]; however, the considerable number of cases of PHPT that fall into this category could make this problematic [9, 19, 25]. Hence, although both patients A and B in our study presented with CCCR values <0.01 (Table 1), this is only suggestive of FHH; a definitive diagnosis of FHH could not be made using this parameter alone. Finally, even when genetic testing is undertaken and a *CASR* variant identified, bioinformatics analysis alone, although helpful, is not in itself conclusive in assigning causality to FHH; we demonstrated in a previous study that there were considerable inconsistencies in the assignment of pathogenicity using bioinformatics approaches to analyse *CASR* variants previously determined by functional analysis to be inactivating and causative of FHH (see reference 18; supplemental data). Taken together, although biochemical parameters such as CCCR can be used as a guide, genetic testing along with bioinformatics analysis and functional assessment of novel variants, as we have shown here and in other studies [17, 18], is fundamental in confirming the diagnosis of FHH.

The L34P variant has a similar profile to another *CASR* variant, G509R, that was examined in previous studies; in that, it failed to produce a mature receptor and it was not activated at any concentration of Ca^++^ ions [17]. In addition, in further studies using confocal microscopy, the G509R receptor was observed trapped in the endoplasmic reticulum and was not present on the cell surface [18]. Given that the L34P receptor fails to dimerize and does not produce a mature, glycosylated receptor, it is likely that it behaves similarly to the G509R receptor, remaining trapped in the endoplasmic reticulum. FHH in this case would be caused by a reduction (by half) of the number of wild-type receptors present on the cell surface as has been shown in a mouse model of FHH [26]. This outcome would be similar to other heterozygously presenting variants that either remain trapped in the endoplasmic reticulum and/or are severely truncated [11, 17, 18, 27]. In order to prevent its accumulation, it is likely that this receptor is recognized by quality control mechanisms in the endoplasmic reticulum and channeled to the proteasome for degradation via the endoplasmic reticulum-associated degradation pathway [28, 29].

## 4. Conclusion

We identified a heterozygous genetic variant in the *CASR* (NM_000388.4:c.T101C:p.Leu34Pro) in a patient presenting with hypercalcaemia. The variant was functionally assessed as inactivating and therefore causative of FHH. Genetic analysis was instigated following careful examination of family history, revealing that the patient's sister had undergone an unsuccessful parathyroidectomy a decade earlier following a provisional diagnosis of PHPT; subsequent genetic analysis of the sister revealed that she also carried the L34P variant heterozygously and therefore can be diagnosed as having FHH rather than PHPT. The study reaffirms the importance of family history evaluation in prompting genetic screening in isolated cases of hypercalcaemia, especially where symptoms and biochemistry make it difficult to differentiate PHPT from FHH. In the case of patient A, unnecessary neck exploratory surgery and parathyroidectomy were avoided. The study, along with others, also highlights the requirement for functional analysis of *CASR* variants, in addition to bioinformatics analysis, in assigning pathogenicity and causality to FHH.

## Figures and Tables

**Table 1:** Patients' biochemical data.

Patient	Date of reading	Serum ionized calcium (pH 7.4) (mmol/L)	Plasma total calcium (corrected) (mmol/L)	Urine calcium excretion (*µ*mol/L GF)	Calcium/creatinine clearance ratio	Serum intact PTH (pmol/L)
A	November 2019	1.43^a^	2.63^b^	8^e^	0.0029	9.2^h^
	November 2021	1.40^a^	2.74^b^	15^f^	0.0056	7.5^h^

B	April 2009 (6 months pre-^*∗*^PTX)	1.39^a^	2.52^c^	22^g^	0.0086	5.3^i^
	September 2009 (immediately post-PTX)	nd	2.44^c^	nd	nd	2.5^j^
	December 2009 (2.5 months post-PTX)	nd	2.52^c^	nd	nd	4.2^i^
	May 2010 (8 months post-PTX)	nd	2.54^c^	nd	nd	3.4^i^
	November 2022 (∼13 years post-PTX)	1.38^a^	2.40^d^	nd	nd	6.5^h^

^*∗*^Parathyroidectomy (PTX) performed on 29^th^ September, 2009; serum ionized calcium reference range: ^a^1.12–1.32 mmol/L; plasma total calcium (corrected) reference ranges: ^b^2.20–2.60 mmol/L, ^c^2.15–2.55 mmol/L, and ^d^2.10–2.60 mmol/L; urine calcium excretion reference ranges: ^e^33–112 *µ*mol/L GF, ^f^47–166 *µ*mol/L GF, and ^g^24–84 *µ*mol/L GF; serum intact PTH reference ranges: ^h^1.6–9.0 pmol/L, ^i^0.7–7.0 pmol/L, and ^j^1.3–6.8 pmol/L; nd = not determined.

**Table 2:** Bioinformatics data for the novel Leu34Pro *CASR* variant.

Variant	SIFT score	PolyPhen-2 HVAR score	CADD Phred score	MetaLR score	Mutation assessor rank score	FATHMM converted rank score	GERP++ RS rank score	SiPhy 29way logOdds rank score
NM_000388.4: c.101T>C: p.(Leu34Pro)	Deleterious (0.001)	Probably damaging (0.997)	Likely benign (27.6)	Deleterious (0.822)	High (0.936)	Deleterious (0.878)	0.782	0.709

Note that as the test variant is novel, the population frequency is not able to be determined. CADD score cutoff for “likely deleterious” is >30 (13).

## Data Availability

The datasets analysed during the current study are available from the corresponding author on reasonable request.

## Abbreviations

ACMG: American College of Medical Genetics
CADD: Combined annotation-dependent depletion
*CASR*: Calcium-sensing receptor gene
CaSR: Calcium-sensing receptor (protein)
CCCR: Calcium creatinine clearance ratio
CT: Computed tomography
ELISA: Enzyme-linked immunosorbent assay
FHH: Familial hypocalciuric hypercalcaemia
GF: Glomerular filtrate
HEK293: Human embryonic kidney 293 (cells)
IP-One: D-myoinositol-1-phosphate
PCR: Polymerase chain reaction
PHPT: Primary hyperparathyroidism
PTH: Parathyroid hormone.

## Supplementary Materials

Supplementary Materials Supplementary Fig. 1: Western blot analysis of the wild type and L34P variant CaSR examined under nonreducing and reducing conditions. HEK293 cells were transfected with the wild type or variant CaSR-FLAG-tagged plasmid and incubated for 48 hrs, and the cells were then lysed and 80 *µ*g of protein with or without reducing agent separated by SDS-PAGE. The gel was blotted and then probed with mouse monoclonal antibody to FLAG-tag followed by goat anti-mouse HRP-conjugated secondary antibody. Protein bands were visualized using ECL chemiluminescence reagent in a Bio-Rad Molecular Imager. ^*∗*^An additional lane is a duplicate of lane 3, WT under reducing (*β*-mercaptoethanol) conditions. Supplementary Fig. 2: Western blot analysis of the same blot shown in Supplementary Fig. 1 reprobed with mouse monoclonal antibody to *α*-tubulin (as a loading control) followed by goat anti-mouse HRP-conjugated secondary antibody. Protein bands were visualized using ECL chemiluminescence reagent in a Bio-Rad Molecular Imager. ^*∗*^An additional lane is a duplicate of lane 3, WT under reducing (*β*-mercaptoethanol) conditions. Note that the weak CaSR signal is from previous exposure to the FLAG-tag antibody.
