# Supplementary material for: Case Presentation: Functional Assessment of a CASR Variant Identified in a Patient with Hypercalcaemia Confirms Familial Hypocalciuric Hypercalcaemia in the Patient and a Sister Previously Misdiagnosed with Primary Hyperparathyroidism
Source: Case Rep Endocrinol. 2024 Feb 3;2024:6652801. doi: 10.1155/2024/6652801 (PMC10858793; doi:10.1155/2024/6652801)
Supplement: Supplementary Materials — Supplementary Fig. 1: Western blot analysis of the wild type and L34P variant CaSR examined under nonreducing and reducing conditions. HEK293 cells were transfected with the wild type or variant CaSR-FLAG-tagged plasmid and incubated for 48 hrs, and the cells were then lysed and 80 µg of protein with or without reducing agent separated by SDS-PAGE. The gel was blotted and then probed with mouse monoclonal antibody to FLAG-tag followed by goat anti-mouse HRP-conjugated secondary antibody. Protein bands were visualized using ECL chemiluminescence reagent in a Bio-Rad Molecular Imager. ∗An additional lane is a duplicate of lane 3, WT under reducing (β-mercaptoethanol) conditions. Supplementary Fig. 2: Western blot analysis of the same blot shown in Supplementary Fig. 1 reprobed with mouse monoclonal antibody to α-tubulin (as a loading control) followed by goat anti-mouse HRP-conjugated secondary antibody. Protein bands were visualized using ECL chemiluminescence reagent in a Bio-Rad Molecular Imager. ∗An additional lane is a duplicate of lane 3, WT under reducing (β-mercaptoethanol) conditions. Note that the weak CaSR signal is from previous exposure to the FLAG-tag antibody. [file 6652801.f1.zip › Ward et al Supplementary Figure 2 (2).pdf]

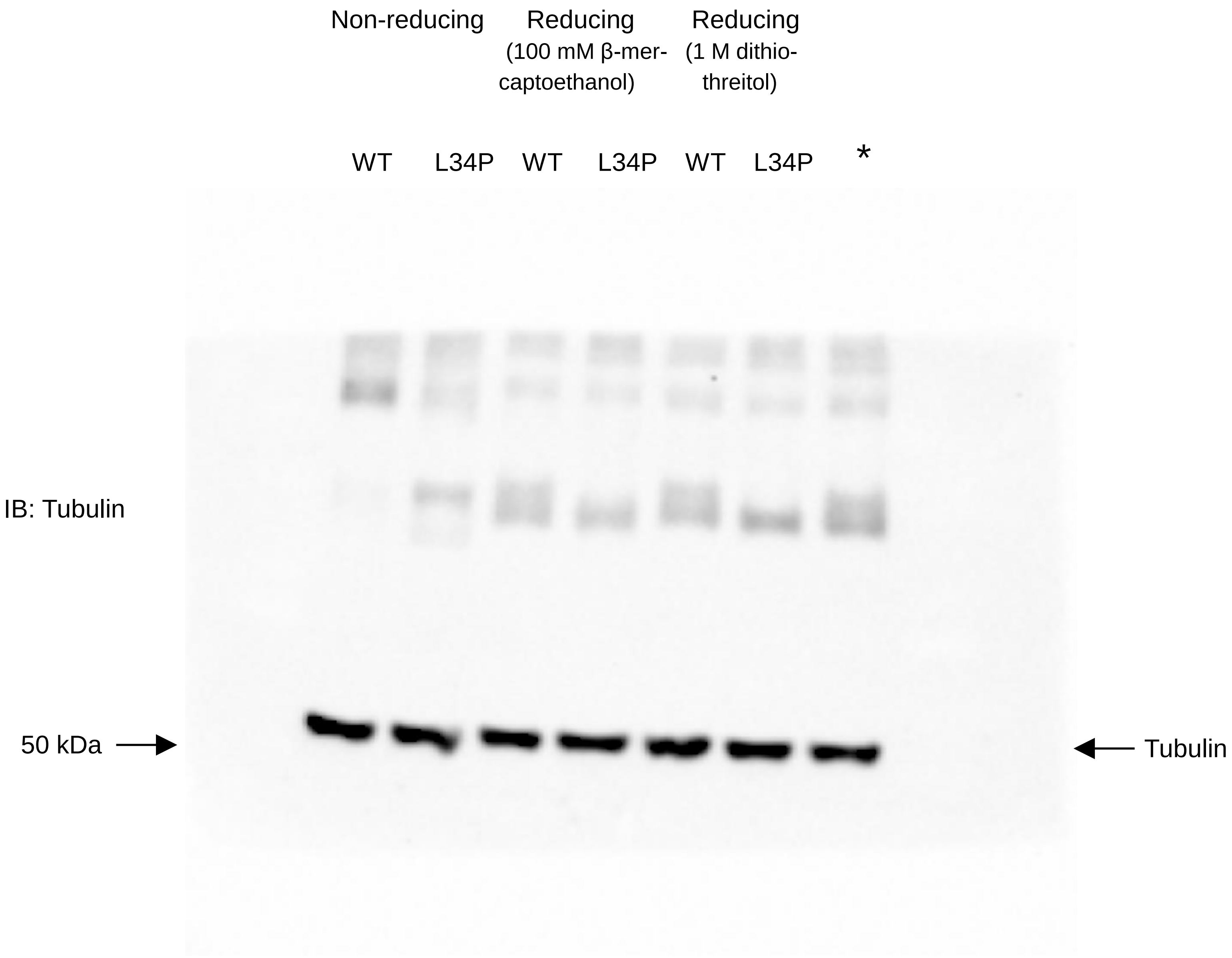

Supplementary Fig. 2 Western blot analysis of the same blot shown in Supplementary Fig. 1 re-probed with mouse monoclonal antibody to α-tubulin (as a loading control) followed by goat anti-mouse HRP-conjugated secondary antibody. Protein bands were visualized using ECL chemiluminescence reagent in a Bio-Rad Molecular Imager. *An additional lane is a duplicate of lane 3, WT under reducing (β-mercaptoethanol) conditions. Note that weak CaSR signal is from previous exposure to FLAG-tag antibody.
